# Early OCT Angiography Changes of Macular Neovascularization in Patients with Exudative AMD Treated with Brolucizumab in a Real-World Setting

**DOI:** 10.1155/2022/2659714

**Published:** 2022-03-25

**Authors:** Anne Rübsam, Saskia Rau, Daniel Pilger, Oliver Zeitz, Antonia M. Joussen

**Affiliations:** ^1^Department of Ophthalmology, Charité University Medicine Berlin, Corporate Member of Freie Universität Berlin, Humboldt-Universität Berlin, Berlin Institute of Health, Berlin, Germany; ^2^Berlin Institute of Health (BIH), Berlin, Germany

## Abstract

**Background:**

To report on the short-term outcome of intravitreal brolucizumab in patients with neovascular age-related macular degeneration (nAMD).

**Methods:**

This is a prospective, interventional, monocentric study on 10 eyes of 10 patients with a treatment-naïve neovascular AMD. Patients were treated according to the HAWK and HARRIER trials. After loading with 3 monthly injections, eyes received an injection 12 weeks after the upload (q12w) or were adjusted to an 8 week interval (q8w), if disease activity was present 8 weeks after the upload. Main outcome measures were the change in central retinal thickness (CRT) assessed by spectral domain optical coherence tomography (SD-OCT), the change in macular neovascularization (MNV) size on optical coherence tomography angiography (OCTA), and the change in best corrected visual acuity (BCVA) 8 and 12 weeks after the upload. We further assessed clinical parameters that predict the treatment response at baseline based on the need of q8w or q12w injections after the upload.

**Results:**

CRT decreased significantly from 461.7 ± 82.9 *μ*m to 343.6 ± 74.3 *μ*m (*p*=0.004) 12 weeks after the upload. The MNV size decreased significantly from 0.85 ± 1.1 to 0.75 ± 1.2 mm^2^ (*p*=0.022). BCVA improved from 0.67 ± 0.4 to 0.55 ± 0.4logMAR but without statistical significance. MNV size in eyes on q12w was considerably smaller compared to that in eyes on q8w (0.54 ± 0.7 mm^2^ vs. 1.98 ± 2.4 mm^2^). The percentage of eyes without any persistent fluid was 70% (7/10 eyes).

**Conclusions:**

Brolucizumab appears to be a valuable tool for the management of patients with nAMD. Furthermore, MNV size at baseline might serve as an early predictor of treatment response.

## 1. Introduction

Antivascular endothelial growth factor (anti-VEGF) therapy has revolutionized the management of neovascular age-related macular degeneration (nAMD) in the last few years. With the widespread adoption of this treatment over the last decade, the blindness attributable to nAMD has been reduced by 50%–72% [1].

Improving or maintaining visual acuity is the main goal for the treatment. The current nAMD standard of care dictates frequent intravitreal anti-VEGF injections, which places a substantial burden on patients, caregivers, and physicians [2, 3]. Multiple treatment regimens have been introduced in recent years to reduce the frequency of anti-VEGF agent dosing while attempting to maintain efficacy comparable to monthly or bimonthly fixed treatment protocols by individualizing therapy [3]. The most practiced individualized approaches are treat-and-extend and pro re nata protocols. Unfortunately, despite relatively good visual outcomes over 1–2 years in prospective studies with these individualized regimens, real-world studies typically show loss of initial visual gains over time [4]. Brolucizumab (Beovu, Novartis), a newly developed anti-VEGF molecule for nAMD treatment, has demonstrated longer durability and improvement in visual and anatomic outcomes in clinical studies, indicating its potential to reduce treatment burden as an important therapeutic tool in nAMD management [5, 6].

Brolucizumab is a humanized single-chain antibody fragment inhibitor of all isoforms of VEGF-A [7]. In comparison to a full antibody or a Fab fragment, it is a single-chain antibody fragment (scFV), an autonomous binding agent that is no longer dependent on a heavy molecular support structure but retains full binding capacity to its target [8]. In comparison to bevacizumab (a full antibody, 149 kDa), aflibercept (a VEGF receptor 1/2–Fc fusion protein, 115 kDa), and ranibizumab (a Fab fragment, 48 kDa), brolucizumab has the smallest molecular weight (26 kDa) [9]. Animal studies have also suggested that brolucizumab's small size might provide better retinal and choroidal penetration and faster systemic clearance [8]. Based on these theoretic assumptions, the biological effect of brolucizumab was postulated to exceed that of the other anti-VEGF agents in terms of effect durability.

Last year, the results of the HAWK and HARRIER approval trials on brolucizumab for treatment-naïve patients with nAMD were published [5]. After a matched phase of 3 loading doses (weeks 0, 4, and 8) for brolucizumab and aflibercept, patients in the brolucizumab group were treated on a q12-week interval, unless they exhibited disease activity, in which case, treatment was adjusted to a permanent q8-week interval. The monitoring of disease activity was assessed at week 16 and at each scheduled q12-week treatment visit during the maintenance phase. During the extension studies, 45.5% and 38.6% of the eyes were functionally maintained and morphologically stable on a 12 weekly regimen of brolucizumab until week 96 [6].

Based on the results of these two clinical trials, we were interested in the short-term outcome of treatment-naïve nAMD patients after brolucizumab therapy in a real-world setting. We assessed disease activity parameters as in the HAWK and HARRIER trials. We further evaluated the macular neovascularization (MNV) size utilizing the ability of optical coherence tomography angiography (OCTA) to noninvasively visualize the MNV directly, both before and after treatment, as a new marker for nAMD activity. We were also interested, if there are clinical parameters, to predict the treatment response at baseline to further discriminate patients at baseline based on the need for 8 weekly or 12 weekly injections after the loading phase.

## 2. Methods

In this institutional, interventional, prospective study, 10 eyes of 10 patients with new-onset neovascular AMD, treated with brolucizumab between May 2020 and July 2021 at the Department of Ophthalmology of the University of Berlin, Charité, Germany, are presented.

All the research and measurements adhered to the tenets of the Declaration of Helsinki; the local ethics committee approved the study. Informed consent was given by the patients' prior study enrollment. Furthermore, patient's consent for publication of photographic material was obtained.

The inclusion criterion was those aged 18 years or older with the presence of a new-onset neovascular AMD, which necessitates therapy with anti-VEGF agents. Exclusion criteria were bilateral wet AMD that necessitates bilateral injections at the same time, AMD in the better-seeing eye (oculus ultimus situation), any ocular condition that was able to interfere with potential visual improvement (i.e., pigment epithelium tear at baseline), signs of any other active retinal disease in the study eye (including the presence of any epiretinal membrane) in particular signs of an active uveitis, patients with status post uveitis, and poor image quality.

### 2.1. Baseline

At baseline, all patients were treatment-naïve. Baseline diagnostic procedures included a best corrected visual acuity (BCVA) assessment in logarithm of the minimum angle of resolution (logMAR), anterior and posterior segment examination, SD-OCT (Heidelberg Spectralis, Heidelberg Engineering, Heidelberg, Germany), fluorescein angiography (FA, Heidelberg Spectralis, Heidelberg Engineering, Heidelberg, Germany), and OCT angiography (OCTA, Heidelberg Engineering, Heidelberg, Germany).

### 2.2. Treatment and Follow-up

Patients were treated on label according to the HAWK and HARRIER protocols with 3 injections of 6 mg brolucizumab in monthly intervals. During the maintenance phase, 8 weeks after the total of 3 injections, a follow-up visit was performed (=16-week interval). Brolucizumab was administered the same day in case of persistent intraretinal or subretinal fluid or an increase in pigment epithelium detachment (PED) on SD-OCT, and patients were rescheduled 8 weeks later for another follow-up visit and possible injection depending on disease activity signs. Patients were observed only and rescheduled for another visit and mandatory injection 4 weeks later (=20-week interval), in case of a dry macula and/or a stable PED on SD-OCT. These patients were also rescheduled 8 weeks later for another follow-up visit and possible injection depending on disease activity signs. We assessed the clinical parameters including BCVA, anterior and posterior segment examination, SD-OCT, and OCTA before each of the three brolucizumab injections (=baseline, visit 1, and visit 2) during the upload phase and at week 16 (=visit 3) and 20 (=visit 4) of the maintenance phase.

### 2.3. OCT Data

Standard settings for OCT recordings were 20° × 20° volume scan and 49 sections at a distance of 122 *μ*m. Central retinal thickness (CRT) in *μ*m was automatically calculated by the device software (software version 5.1.2.0) as the distance from the retinal pigment epithelium (RPE) to the inner limiting membrane (ILM) at the highest point within a circle of 1 mm radius centered on the fovea. We further assessed the PED height in *μ*m by manual measurement of the distance between the RPE and the Bruch membrane at the highest concavity and the presence of intraretinal or subretinal fluid.

For the OCTA examinations, 70,000 A-scans were acquired and a 15° × 15° scan angle protocol was used to gain a total of 261 B-scans with a resolution of 5.7 *μ*m/pixel. The standard OCTA viewing module (software version 6.9.5.0), and its associated automatic segmentation of the retinal layers, was applied to derive the en face slabs for each vascular plexus. The MNV size was manually marked and sized in square millimetres with the evaluation tool of the device.

### 2.4. Main Outcome Measures

According to the need for an injection at either week 16 or week 20 of the maintenance phase, patients were further divided into 2 subgroups:

1. 8-week injection interval (2 eyes), and
2. 12-week injection interval (8 eyes).

Main outcome measures were defined as reduction of CRT, BCVA, and MNV size at week 16 and 20. Secondary outcome measures were safety, percentage of eyes achieving complete resolution of fluid accumulation (intraretinal, subretinal, and/or sub-RPE fluid) at weeks 16 and 20, and clinical parameters that predict the treatment outcome (necessity for 8 weekly injections or 12 weekly injections after the upload).

### 2.5. Statistical Analysis

The mean ± SEM and statistically significant differences are reported. Values for CRT, BCVA, and MNV size were compared to baseline at the different time points using paired two-tailed Student *t*-tests. Because of the small sample size, we also performed a Wilcoxon signed-rank test to account for a non-Gaussian distribution and compared the significance levels of the parametric and nonparametric tests. The results were comparable and allowed the interpretation of the parametric findings. To ease the interpretation, we are only reporting the parametric test results. Frequencies were compared with Fisher exact tests. A *p*-value inferior to 0.05 was considered significant. The statistical analysis was performed using GraphPad Prism (GraphPad Software, San Diego, CA, USA).

## 3. Results

A total of 10 eyes from 10 patients (5 male and 5 female) with neovascular AMD were included in the study with a mean age of 81 ± 5.7 years (Table 1). The AMD was unilateral in 9 (90%) patients and bilateral in 1 (10%) patient, with a quiescent MNV in one eye and an active MNV in the fellow eye.

The MNV type was determined by assessing the pattern and time point of leakage on FA and confirmed by the presence of MNV on OCT (intraretinal, below, or above the RPE). FA was performed at baseline in 9/10 (90%) patients. One patient with a decrease in renal function did not receive an FA. In this patient noninvasive OCTA was used to confirm the presence of MNV. OCTA images are available for all patients. Most patients showed an angiographic type 1 MNV at baseline (Table 1).

### 3.1. Central Retinal Thickness

**Figure 1:**
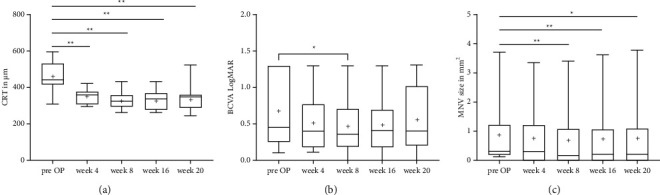
Boxplot graph representing changes in central retinal thickness (CRT), best corrected visual accuity (BCVA), and macular neovascularization (MNV) size after therapy with three injections of brolucizumab in patients with exudative age-related macular degeneration (AMD). The figure shows the difference in change in (a) CRT in *μ*m, (b) BCVA in logMAR, and (c) MNV size in mm^2^ after three monthly loading doses of brolucizumab (at baseline, week 4, and week 8), 8 weeks (week 16), and 12 weeks (week 20) after the upload. The boxplot graph is depicting the median (line) and the mean (cross) values for each individual time point. ^*∗*^*p* < 0.05, ^*∗∗*^*p* < 0.01.

Mean CRT decreased significantly from baseline to the last visit (*p*=0.004; for details Table 2, Figure 1 and supplemental file (available (here))). In detail, mean CRT decreased in all patients significantly after the first injection (*p*=0.003) and decreased further after the second injection (*p*=0.001). After the 8-week interval (week 16, visit 3), CRT increased slightly but still decreased compared to baseline with statistical significance (*p*=0.002). At 12 weeks after the upload (week 20, visit 4), CRT increased further slightly but decreased compared to baseline with statistical significance (*p*=0.004). Patients with a q12w interval (group 1) showed a better CRT reduction after brolucizumab treatment compared to patients on a q8w interval (group 2). The detailed CRT values for the q12w patients are as follows: baseline: 460.2 ± 97.6 *μ*m; visit 1 : 360.5 ± 50.5 *μ*m; visit 2 : 311.6 ± 37.2 *μ*m; visit 3 : 309.3 ± 39.5 *μ*m; and visit 4 : 339.8 ± 83.3 *μ*m. The detailed values for the q8w patients are as follows: baseline: 467.5 ± 45.9 *μ*m; visit 1 : 395 ± 55.6 *μ*m; visit 2 : 393 ± 53.7 *μ*m; visit 3 : 429.5 ± 2.1 *μ*m; and visit 4: not applicable.

### 3.2. Best Corrected Visual Acuity

BCVA increased in all patients from baseline to the last visit but without statistical significance (*p*=0.112; Table 2, Figure 1). In detail, mean BCVA increased already after the first injection (*p*=0.068) and increased further significantly after the second injection (*p*=0.044). At week 16, BCVA decreased slightly (*p*=0.080). At week 20, BCVA decreased further (*p*=0.112). Patients in group 1 showed a greater increase in visual acuity after brolucizumab treatment compared to patients in group 2. The detailed BCVA values for the q12w patients are as follows: baseline: 0.75 ± 0.5; visit 1 : 0.56 ± 0.5; visit 2 : 0.52 ± 0.5; visit 3 : 0.52 ± 0.5; and visit 4 : 0.55 ± 0.4. The detailed values for the q8w patients are as follows: baseline: 0.35 ± 0.2; visit 1 : 0.3 ± 0.1; visit 2 : 0.25 ± 0.1; visit 3 : 0.3 ± 0.1; and visit 4: not applicable.

### 3.3. Macular Neovascularization Size

MNV size decreased significantly in all patients from baseline to the last visit (*p*=0.022, Table 2, Figure 1). In detail, mean MNV area decreased significantly already after the first injection (*p*=0.050) and decreased further after the second injection (*p*=0.001). At week 16, MNV size increased slightly but still decreased with statistical significance compared to baseline (*p*=0.002). At week 20, MNV size increased further but still decreased with statistical significance compared to baseline (*p*=0.022). MNV size in group 1 decreased during the upload and increased again during the maintenance phase to nearly pretreatment levels at the 12-week interval, whereas patients in group 2 showed an initial further increase in MNV size and a slight decrease to pretreatment size 8 weeks after the upload. The detailed values for the change in mean MNV area for q12w patients are as follows: baseline: 0.54 ± 0.7 mm^2^; visit 1 : 0.49 ± 0.7 mm^2^; visit 2 : 0.43 ± 0.6 mm^2^; visit 3 : 0.43 ± 0.6 mm^2^; and visit 4 : 0.55 ± 1 mm^2^. The detailed values for the q8w patients are as follows: baseline: 1.98 ± 2.4 mm^2^; visit 1 : 2.1 ± 2.5; visit 2 : 1.85 ± 2.2; visit 3 : 1.98 ± 2.4; and visit 4 :  not applicable.

### 3.4. Anatomic Outcome and Treatment Interval

**Figure 2:**
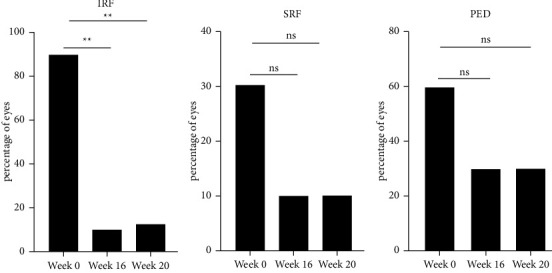
Percentage of eyes with intraretinal fluid (IRF), subretinal fluid (SRF), and pigment epithelium detachment (PED) size after therapy with three injections of brolucizumab in patients with exudative age-related macular degeneration (AMD). The figure shows the change in IRF, SRF, and PED at baseline (week 0), 8 weeks (week 16), and 12 weeks (week 20) after three monthly loading doses of brolucizumab. ^*∗∗*^*p* < 0.01.

The percentage of eyes without any persistent fluid was 80% (8/10 eyes) at week 16 and 70% (7/10 eyes) at week 20. 80% (8/10 eyes) of the patients were on a q12w interval 12 weeks after the upload. Of the two patients on a q8w interval, one patient had persistent fluid after the upload and one patient had recurrent fluid at this time point. Brolucizumab showed the greatest effect on intraretinal fluid (IRF), which resolved in 77.5% of the patients. At baseline, IRF was present in 90% of the patients, at week 16, it was present in 10% of the patients (*p* < 0.001,*p*=0.001), and at week 20, it was present in 20% of the patients (*p*=0.005). The subretinal fluid (SRF) accumulation resolved in 2/3 of the patients but without statistical significance (baseline: 30%, week 16 and 20 : 10% each, *p*=0.369). The sub-RPE fluid resolved in half of the patients with a PED at baseline (baseline 60%, week 16 and 20 : 30% each, *p*=0.582; Figure 2).

### 3.5. Safety

In our study, no patients experienced any endophthalmitis, RPE rip, or extensive macula bleeding. We observed one case of intraocular inflammation related to the brolucizumab injection 7 days after the 5th injection. The patient presented with sudden onset, painless blurring of vision and floaters. Visual acuity decreased from 0.0 to 0.2 logMAR. Anterior segment examination was normal. In the posterior segment, intermediate uveitis with snowballs and vitreous haze were present. Vasculitis or vascular occlusion was ruled out by FA. Patient was started on oral steroids, which were tapered gradually. Two weeks later, visual acuity increased to 0.0 logMAR again and inflammation ceased.

## 4. Discussion

Recently, brolucizumab was approved as a new anti-VEGF agent for intravitreal administration in patients with nAMD. However, to date, only limited data of its use in a real-world setting are available [10–18]. Herein, we report early experiences with the brolucizumab use in clinical routine in a single center in Germany. Our results indicate a favorable outcome of treatment-naïve nAMD patients 16 and 20 weeks after therapy initiation with brolucizumab. We found a significant reduction in CRT along with an increase in BCVA, albeit without statistical significance at week 16 or 20. We further investigated the pathophysiology behind this treatment effect and found MNV size to decrease significantly at weeks 16 and 20. As a result of the favorable treatment effect, 80% of the patients could be maintained on a q12w dosing interval. Interestingly, we found morphological parameters and functional outcome to decline at the 8- and 12-week intervals after the upload (weeks 16 and 20). This indicates a recurrence of the MNV activity and the necessity for a subsequent brolucizumab injection.

Our results reproduce the favorable outcome of the phase-III HAWK and HARRIER trials of treatment-naïve nAMD patients in a real-world setting [5, 6]. Both studies showed that at week 16, 6 mg brolucizumab demonstrated a significant decrease in CRT (HAWK: −161.4 *μ*m; HARRIER: −174.4 *μ*m) [5]. In our study, CRT decreased to a slightly smaller extent but also significantly by 128 *μ*m. Of note, baseline CRT was similar in our study compared to the two trials (461.7 *μ*m versus 463.1 *μ*m in HAWK and 473.6 *μ*m in HARRIER). In our study, the effect of brolucizumab was greatest on the IRF, followed by PED and SRF. In HAWK and HARRIER trials, the therapy effect was greatest on the SRF, followed by IRF and PED. Of note, the baseline characteristics between the HAWK and HARRIER studies and our study differed in the way that in our study, most patients presented with IRF (90%) compared to SRF (30%) or PED (60%), whereas in both pivotal trials, most patients presented with SRF (69.4 and 67.7%) compared to IRF (53.9 and 40.3%) or PED (46.7 and 33.8%). Regarding the treatment interval during the maintenance phase, 76% of the eyes in HAWK and 77% of the eyes in HARRIER were functionally maintained and morphologically stable on q12w interval at a dose of 6 mg at week 16, and this was maintained in over half (51–56%) of the eyes throughout until week 48 [5]. This is similar to our results with 80% of the eyes on a q12w interval at week 16. In accordance with the anatomical improvement, we demonstrated a significant improvement in visual acuity during the upload phase, which declined slightly during the maintenance phase in our study. This differs to the pivotal trials, where the initial increase in BCVA after the upload could be maintained at weeks 16 and 20 and throughout one year [5]. The slight decrease in visual acuity in our study is caused by one patient with a significant worsening in VA at week 20 due to recurrent fluid. In all other patients, the observed gain in VA could be maintained during the maintenance phase as seen in the HAWK and HARRIER trials.

There are only few other studies on the real-world experience with brolucizumab for treatment-naïve patients with nAMD [12, 15–18]. Of these studies, two studies had a different treatment protocol compared to the HAWK and HARRIER trials [15, 17] and two studies a shorter follow-up period compared to our study [12, 18]. Our results are comparable to the results reported in the REBA study on 25 treatment-naïve eyes with nAMD with a similar distribution of MNV subtypes. Here, the authors also show a significant reduction in CRT (*p*=0.021) and furthermore, a significant increase in BCVA (*p*=0.011). Although the percentage of patients on a q12w interval (68% vs. 80% in our study) and the percentage of patients with a dry macula 12 weeks after the switch (48% vs. 70%) was lower compared to our study. In another study by Matsumoto et al., CRT and BCVA improved significantly (both *p* < 0.001) in all 36 eyes at week 12 after baseline [12]. A dry macula was achieved even in 34/36 eyes (94.4%) [12]. The results are comparable to our results only in a limited extent because the authors already assessed the clinical parameters 4 weeks after the upload (week 12), which differed from the assessment in the brolucizumab pivotal trials and from our study. Furthermore, they included patients only with type 1 MNV, of which 53% of the patients demonstrated with polypoidal choroidal vasculopathy (PCV), which is more common in the Asian population than in Caucasians [19]. PCV resembles neovascular AMD in many of its morphological features [20, 21]. However, the clinical course of PCV is more stable and visual outcomes are more favourable to those of AMD [20, 22–24], which might explain the more favourable outcome compared to our study.

**Figure 3:**
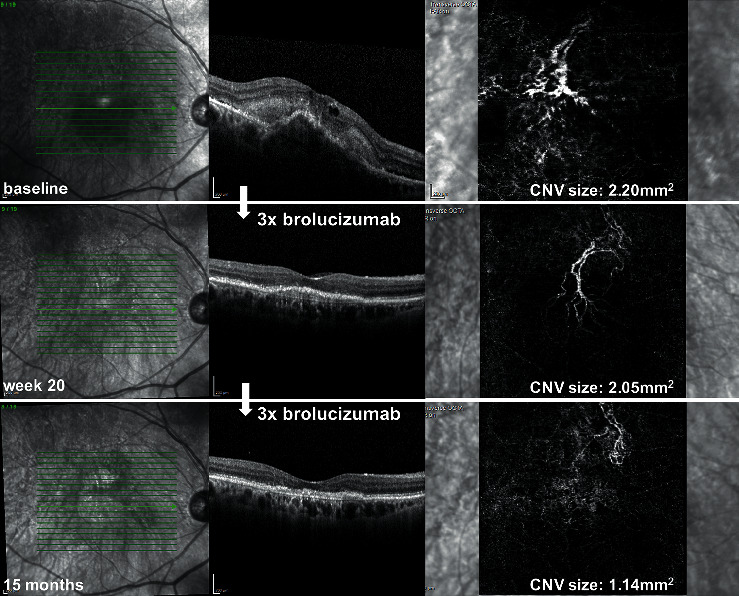
Treatment response after brolucizumab therapy assessed by optical coherence tomography (OCT) and optical coherence tomography angiography (OCTA) at baseline, 12 weeks after the upload (week 20), and at the end of the follow-up period. A 91-year-old woman with type 1 macular neovascularization (MNV) on OCTA with intraretinal fluid (IRF) and a fibrovascular pigment epithelium detachment on OCT with a complete regression of IRF and a reduction in PED height 12 weeks after three monthly loading doses of brolucizumab (week 20). The macula stayed dry and PED height decreased further after 3 more injections every 12 weeks at the end of the follow-up period (15 months). The MNV area assessed by OCTA decreased at week 20 and decreased further at 15 months.

We furthermore investigated the pathophysiology behind the treatment effect, utilizing the ability of OCTA to noninvasively visualize the MNV before and after treatment. In the literature, there is evidence that the change in MNV size serves as a marker for disease activity and thus fluid accumulation. Bailey et al. found that in eyes with pre-exudative AMD, an MNV area growth rate of 20% per month is associated with a high risk of developing exudation [25]. Furthermore, in other studies, anti-VEGF treatment reduced the MNV size along with a reduction in exudation [14, 18, 26, 27]. Herein, we could demonstrate that the MNV size significantly decreased in our study cohort under brolucizumab therapy along with a decrease in exudation (Figure 3). This is in line with two other studies investigating the change in MNV area under brolucizumab therapy, which investigated the effect after one to three injections with a maximum follow-up time of 7 days after the upload [14, 18]. Interestingly, during the maintenance phase in our study, MNV size increased again, suggesting that the brolucizumab effect diminishes and yet another injection is necessary. Interestingly, MNV size serves as an early clinical parameter for treatment response to brolucizumab as it only decreased in patients with a better response to brolucizumab, which could be maintained on a q12w interval. In patients with a poorer response to brolucizumab, which needed a follow-up injection at q8w after the upload, the MNV size did not decrease after therapy. Furthermore, patients on a q12w interval had a notably smaller MNV size at baseline compared to patients on a q8w interval (0.54 ± 0.7 mm^2^ vs. 1.98 ± 2.4 mm^2^). Our observed high percentage of patients that could be maintained on a q12w dosing interval seems to confirm the theoretical advantage of brolucizumab in terms of its pharmacokinetics with its low molecular weight with subsequent better tissue penetration as well as higher molar concentration [8]. At a dose of 6 mg, its equivalent molar dose is approximately 10 times greater than aflibercept and approximately 20 times greater than bevacizumab and ranibizumab [9].

We performed 40 brolucizumab injections during the study period and 59 injections in total within the follow-up period of 11 months (range 7–15 months). Up to date, we observed one case of brolucizumab-related intraocular inflammation (IOI) in an 86-year-old woman with a type 1 MNV with no history of uveitis or systemic autoimmune disease, which presented with a decreased vision from 0.1 to 0.2 logMAR and floaters 8 days after her 5th injection. On examination, snowballs and vitreous cells resembling intermediate uveitis were detectable. There were no signs of occlusive vasculitis on FA. She was treated with oral steroids, and all inflammatory signs resolved and vision increased to 0.1 logMAR. Nevertheless, there are significant concerns about IOI in the literature, including retinal occlusive vasculitis after intravitreal brolucizumab, which can result in permanent loss of vision [28–33]. Novartis thoroughly investigated this phenomenon and reported an incidence rate of 10.67 per 10 000 injections for retinal vasculitis and/or retinal vascular occlusion and a 4% rate for IOI (as of August 2020) [6, 34]. The largest case series reported to the American Society of Retina Specialists [35] concluded that this inflammation could develop weeks after the last brolucizumab injection (mean of 25 days (range, 3–63 days)) [30]. Although a few eyes in this series were asymptomatic (8%) or minimally symptomatic (blurry vision 62%, floaters 46%, pain 31%, and redness 19%), some eyes had significant vision loss [30]. Most eyes had evidence of retinal vascular occlusion and/or retinal ischemia on examination and/or imaging [30]. Treatment approaches varied with almost all patients being treated with topical steroids, almost half of them with systemic steroids, 20% received intravitreal steroids and few patients underwent vitrectomy [30]. Although the exact mechanism of these findings remains unclear, the ASRS Research and Safety in Therapeutics (ReST) Committee recommends a careful evaluation of the anterior and posterior segments for any signs of active inflammation prior to any brolucizumab injection [30]. Appropriate informed consent should be obtained, and patients should be advised to return for prompt evaluation with changes. Because of the potentially severe nature of the consequences of retinal vasculitis secondary to brolucizumab, we are cautious when considering injection of brolucizumab in monocular patients or when bilateral injections are being contemplated.

The major strengths of our study are its prospective design, the standardized treatment protocol and follow-up, and the inclusion of only treatment-naïve patients, which allows drawing conclusions on the anatomic efficacy and durability of the brolucizumab treatment and allows a direct comparison with the pivotal studies in a real-world scenario. Limitations of our study include the single-center design, the small number of patients, and the short follow-up of only one year. Thus, long-term outcomes and the efficacy of intravitreal brolucizumab for other nAMD subtypes still needs to be evaluated.

In conclusion, our results show that brolucizumab appears to be a valuable tool for the management of patients with neovascular AMD. These patients might have an anatomic improvement, with reductions of CRT, MNV size, and retinal exudation with an increase in visual acuity, while the treatment intervals could be extended.

## Figures and Tables

**Table 1:** Baseline characteristics of 10 treatment-naïve neovascular age-related macular degeneration patients.

No of eyes	10
Men/women	5/5
Age, years	80.9 ± 6
MNV type	9/10
Type 1	7
Type 2	1
Type 3	1
Central retinal thickness, *μ*m	461.7 ± 82.9
PED, *μ*m	202.5 ± 82.7
BCVA, logMAR	0.67 ± 0.4
MNV size, mm3	0.85 ± 1.1

BCVA = best corrected visual acuity; MNV = macular neovascularization; OCTA = optical coherence tomography angiography, PED = pigment epithelium detachment.

**Table 2:** Mean change in central retinal thickness, macular neovascularization size, and best corrected visual acuity of 10 treatment-naïve neovascular age-related macular degeneration patients at baseline and during follow-up.

Visit	Mean CRT + SD (*μ*m) *p*-value^*∗*^	Mean MNV size + SD (mm2) *p*-value^*∗*^	Mean BCVA + SD (logMAR) *p*-value^*∗*^
Baseline	461.7 ± 82.9	0.85 ± 1.1	0.67 ± 0.4
4 weeks	351.0 ± 39.1 *p*=0.003	0.75 ± 1.0 *p*=0.050	0.51 ± 0.4 *p*=0.068
8 weeks	327.9 ± 48.1 *p*=0.001	0.70 ± 1.0 *p*=0.001	0.47 ± 0.4 *p*=0.044
16 weeks	333.3 ± 58.4 *p*=0.002	0.74 ± 1.1 *p*=0.002	0.478 ± 0.4 *p*=0.080
20 weeks	343.6 ± 74.3 *p*=0.004	0.75 ± 1.2 *p*=0.002	0.55 ± 0.4 *p*=0.112

BCVA = best corrected visual acuity; CRT = central retinal thickness; logMAR = logarithm of minimal angle of resolution; MNV = macular neovascularization; SD = standard deviation ^*∗*^two-tailed Student *t*-test of visit (week) - baseline.

## Data Availability

The clinical data used to support the findings of this study are included within the supplementary information file(s).

## Supplementary Materials

Supplementary Materials (a) Consort-2010-Checklist-Beovu Manuscript: Supplemental Word file with the CONSORT clinical trial checklist. (b) Supplemental file clinical data. Supplemental Word file with an overview over the included study patients. Date of birth, gender, type of MNV at baseline and central retinal thickness, best corrected visual acuity, MNV size at baseline, before each injection and 16 and 20 weeks after the initial three injections.
