# Supplementary material for: Early OCT Angiography Changes of Macular Neovascularization in Patients with Exudative AMD Treated with Brolucizumab in a Real-World Setting
Source: J Ophthalmol. 2022 Mar 25;2022:2659714. doi: 10.1155/2022/2659714 (PMC8975682; doi:10.1155/2022/2659714)
Supplement: Supplementary Materials — (a) Consort-2010-Checklist-Beovu Manuscript: Supplemental Word file with the CONSORT clinical trial checklist. (b) Supplemental file clinical data. Supplemental Word file with an overview over the included study patients. Date of birth, gender, type of MNV at baseline and central retinal thickness, best corrected visual acuity, MNV size at baseline, before each injection and 16 and 20 weeks after the initial three injections. [file 2659714.f1.zip › 2659714.f1/Supplemental file clinical data_revised.docx]

|  |  |  |  |  |  |  |  |
| --- | --- | --- | --- | --- | --- | --- | --- |
|  |  |  |  |  |  |  |  |
| Name | DOB | current treatment interval | no injections until 05/21 | follow up until 05/21 in months | age | Date FA | type of MNV on FA |
| Beovu 001 | 06.05.30 | 12 w | 6 | 12 | 91 | 04.05.20 | No FA |
| Beovu 002 | 16.05.50 | 12 w | 5 | 7 | 71 | 24.09.20 | Type 1 |
| Beovu 003 | 25.09.35 | 12 w | 6 | 11 | 86 | 29.05.20 | Type 2 |
| Beovu 004 | 24.12.35 | 4 w | 4 | 9 | 86 | 26.08.20 | Type 1 |
| Beovu 005 | 10.12.36 | 12 w | 5 | 8 | 85 | 02.09.20 | Type 1 |
| Beovu 006 | 06.10.38 | 12 w | 7 | 11 | 83 | 05.06.20 | Type 1 |
| Beovu 007 | 12.04.46 | 12 w | 6 | 10 | 75 |  | Type 3 |
| Beovu 008 | 13.03.39 | 10-12 w | 8 | 11 | 82 | 03.06.20 | Type 1 |
| Beovu 009 | 07.02.37 | 12 w | 4 | 10 | 84 | 15.07.20 | Type 1 |
| Beovu 010 | 28.10.45 | Switched to eylea | 8 | 11 | 76 | 11.06.20 | Type 1 |
|  | **mean** |  | **59** | **10** | 81,9 |  |  |
|  | **SD** |  | **5,9** |  | 5,769748695 |  |  |
|  |  |  |  |  |  |  |  |
|  |  |  |  |  |  |  |  |
|  |  |  |  |  |  |  |  |
|  |  |  |  |  |  |  |  |
|  |  |  |  |  |  |  |  |
|  |  |  |  |  |  |  |  |

| 1. injection | |  |  | 2 injection | |  |  | 3 injection | |  |  |  |
| --- | --- | --- | --- | --- | --- | --- | --- | --- | --- | --- | --- | --- |
| pre OP | | | | 4 weeks | | | | 8 weeks |  |  |  |  |
| date | MNV size | BCVA | CRT | date | MNV size | BCVA | CRT | date | MNV size | BCVA | dry | CRT |
| 07.05.20 | 2,07 | 1,3 | 425 | 04.06.20 | 2,07 | 1,3 | 364 | 02.07.20 | 1,97 | 1,30 | y | 265,00 |
| 09.10.20 | 0,17 | 0,25 | 594 | 11.11.20 | 0 | 0,2 | 381 | 11.12.20 | 0,00 | 0,10 | y | 363,00 |
| 29.05.20 | 0,11 | 1,3 | 447 | 30.06.20 | 0,02 | 0,6 | 359 | 31.07.20 | 0,03 | 0,50 | y | 301,00 |
| 26.08.20 | 0,32 | 0,1 | 380 | 02.10.20 | 0,32 | 0,1 | 331 | 30.10.20 | 0,17 | 0,00 | y | 321,00 |
| 11.09.20 | 0,16 | 0,4 | 431 | 09.10.20 | 0,16 | 0,1 | 413 | 06.11.20 | 0,05 | 0,20 | y | 326,00 |
| 11.06.20 | 3,69 | 0,2 | 500 | 09.07.20 | 3,32 | 0,2 | 355 | 07.08.20 | 3,39 | 0,20 | y | 355,00 |
| 21.07.20 | 0,93 | 1,3 | 577 | 21.08.20 | 0,93 | 1,3 | 422 | 18.09.20 | 0,79 | 1,30 | y | 354,00 |
| 03.06.20 | 0,25 | 1 | 519 | 02.07.20 | 0 | 0,5 | 296 | 30.07.20 | 0,00 | 0,40 | y | 261,00 |
| 15.07.20 | 0,53 | 0,4 | 309 | 11.08.20 | 0,48 | 0,4 | 300 | 14.09.20 | 0,46 | 0,40 | y | 302,00 |
| 22.06.20 | 0,28 | 0,5 | 435 | 20.07.20 | 0,28 | 0,4 | n.e. | 19.08.20 | 0,15 | 0,30 | n | 431,00 |
| **mean** | 0,851 | 0,675 | 461,7 |  | 0,758 | 0,51 | 351 |  | 0,701 | 0,47 |  | 327,9 |
| **STDV** | 1,100667525 | 0,468641654 | 82,9205041 |  | 1,042293625 | 0,425323406 | 39,07684737 |  | 1,065339852 | 0,43829214 |  | 48,11953865 |
| **median** | 0,30 | 0,45 | 441,00 |  | 0,30 | 0,40 | 357,00 |  | 0,16 | 0,35 |  | 323,50 |
|  |  |  |  |  |  |  |  |  |  |  |  |  |
| MNV size >0,5 | 1 of 8 dry |  |  |  |  |  |  |  |  |  |  |  |
| MNV size < 0.5 | 1 of 2 wet at 12 week interval | | |  |  |  |  |  |  |  |  |  |

| 16 week |  |  |  |  | 20 week |  |  |  |  |
| --- | --- | --- | --- | --- | --- | --- | --- | --- | --- |
| 8 week post 3. IVOM; | | | | | 12 weeks post IVOm | | | | |
| date | MNV size | BCVA | dry | CRT | date | MNV size | BCVA | CRT | dry |
| 31.08.20 | 2,12 | 1,30 | y | 265,00 | 05.10.20 | 2,12 | 1,10 | 245,00 | y |
| 09.02.21 | 0,00 | 0,10 | y | 351,00 | 11.03.21 | 0,00 | 0,20 | 351,00 | y |
| 29.09.20 | 0,02 | 0,40 | y | 261,00 | 23.10.20 | 0,07 | 0,40 | 274,00 | y |
| 04.01.21 | 0,26 | 0,20 | y | 351,00 | 01.02.21 | 0,26 | 0,20 | 351,00 | y |
| 04.01.21 | 0,02 | 0,00 | y | 326,00 | 01.02.21 | 0,01 | 0,00 | 330,00 | y |
| 07.10.20 | 3,62 | 0,20 | no | 428,00 | 06.11.20 | 3,77 | 0,30 | 374,00 | y |
| 17.11.20 | 0,74 | 1,30 | y | 348,00 | 15.12.20 | 0,74 | 1,30 | 342,00 | no |
| 28.09.20 | 0,00 | 0,50 | y | 274,00 | 27.10.20 | 0,00 | 1,00 | 522,00 | no |
| 18.11.20 | 0,47 | 0,40 | y | 298,00 | 18.12.20 | 0,47 | 0,40 | 298,00 | y |
| 02.11.20 | 0,15 | 0,40 | no | 431,00 | s.p. IVI 4 weeks ago | | |  |  |
|  | 0,74 | 0,48 |  | 333,3 |  | 0,826666667 | 0,544444444 | 343 |  |
|  | 1,139394576 | 0,435430821 |  | 58,36445836 |  | 1,220109285 | 0,437444882 | 74,30417963 | #DIV/0! |
|  | 0,21 | 0,40 |  | 337,00 |  | 0,26 | 0,40 | 342,00 |  |
|  |  |  | 8/10 dry macula | |  |  |  |  |  |
|  |  |  |  |  |  |  |  | 12 week interval 8/10 | |
|  | 1,00 |  |  |  |  |  |  |  |  |
|  |  |  |  |  |  |  |  |  |  |
